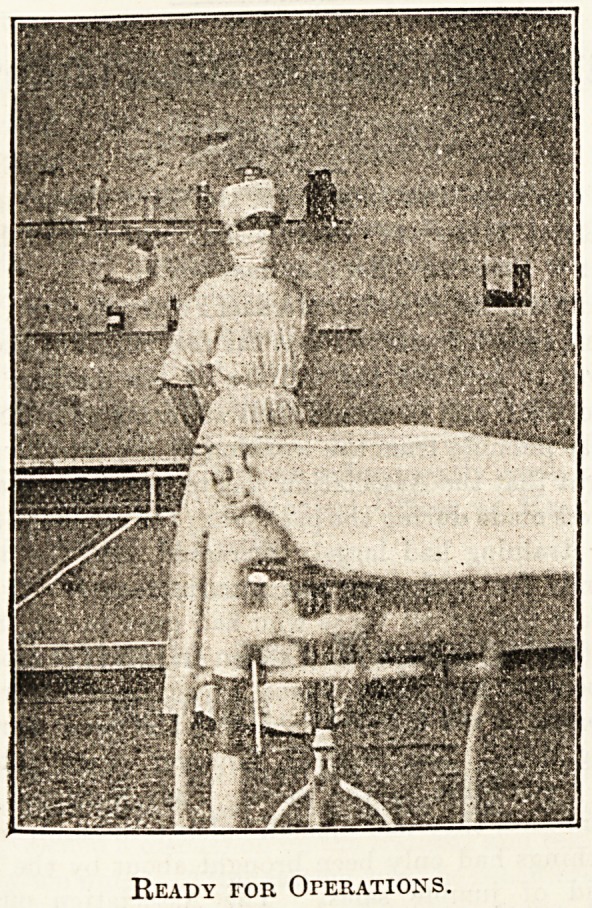# "The Hospital" Nursing Section

**Published:** 1906-05-12

**Authors:** 


### The Hospital.

### Nursing Section.

Contributions for "The Hospital," should be addressed to the Editor, "The Hospital" Nursing Section, 28 & 29 Southampton Street, Strand, London, W.C.

No. 1,024 —Vol. XL. SATURDAY, MAY 12 1906.

### Notes on News from the Nursing World.

### THE PRINCESS ROYAL AND THE CHILDREN.

The sun which shone down upon the ceremony of laying the foundation-stone at the extension of the Bolingbroke Hospital on Saturday last was heartily welcomed by the staff, who had been looking forward to the auspicious day all the week. Behind the visitors, exactly opposite the platform, were a row of boxes raised from the ground, which were utilised by the domestics, whilst the nurses were given the last row of chairs. The advantage of this was that when the Princess Royal was laying the stone the nurses, having no one behind them, were able to stand up and so obtain a splendid view of all that went on. They were, of course, particularly interested in the presentation by the matron, Miss Russell, of a bouquet of pink roses tied with wide pink ribbon to her Royal Highness, and those whose duty was amongst the children were surprised when later on they returned to the wards to find each little person rejoicing in a fragrant rose exactly similar to those which they had last seen on the platform. Then they realised what had not been generally known, that before they left the building the Princess Royal and her husband had, at their own wish, gone through the wards, and the former had pulled out some of the flowers from her bouquet in order to give one to each little sufferer.

### QUEEN VICTORIA'S JUBILEE INSTITUTE.

We learn from the report for 1905 submitted to Her Majesty by the Council of Queen Victoria's Jubilee Institute for nurses, that the Council were obliged last year, in order to meet the necessary expenditure, to withdraw £1,600 from capital. But the special committee initiated by Queen Alexandra will, it is hoped, be able to make good the deficiency. It is noteworthy that nearly half the income is devoted to the training of nurses, and another quarter to inspection and superintendence, in order to maintain the standard of nursing work throughout the country. The total number of associations now affiliated to the Institute employing Queen's Nurses is 694, and the number of Queen's Nurses employed on January 1 was 1,313. Both figures show a considerable increase on the previous year.

### DEATH OF AN ARMY MATRON.

A cablegram has been received at the War Office announcing the death of Sister Lucie Todd, acting matron of the Military Hospital at Middelburg, Cape Colony. One of the first army sisters to sail to the Cape on the outbreak of the South African War, Miss Todd was sent to the Mooi River base, and shared with a few others the severe strain entailed by the nursing of the stream of wounded which flowed from a succession of engagements now famous in history. From Mooi River she was transferred to Elandsfontein and Pretoria, returning later to her first station as acting matron, and it was in a similar capacity that the last months of her life were spent at Middelburg. With characteristic devotion she fulfilled her responsible duties almost up to the last, and the end came on April 29. Miss Todd was trained at Addenbrooke's Hospital, Cambridge, and Charing Cross Hospital, and completed her experience with a midwifery certificate and some months in a fever hospital.

### PROGRESS OF A GREAT TRAINED NURSES' INSTITUTION.

The report of Guy's Hospital Trained Nurses' Insttution for 1905, which, as our readers know, this important agency attained its majority, is full of interest. The year began and ended with a staff of 106 nurses, the number slightly varying during its progress. As on former occasions, the Institution maintained the training of 42 probationers in Guy's Hospital at a cost of £580 for board and lodging. Applications for nurses were received in 1,171 cases, as compared with 1,149, and a feature of the patients' maladies is the considerable increase in medical cases. About half the applications for nurses in private cases came from London and the suburbs, and the other half from the provinces and abroad. Of the twenty-four nurses who ceased to be members of the Institution, four returned to Guy's Hospital on their appointment as sisters, and the majority of the others became sisters in other hospitals or joined other nursing institutions. The number of nurses who shared in the bonus of £2,094 was 53, the bonus being apportioned at the rate of £6 per share, and invested in the Royal National Pension Fund for their future maintenance. The total payment to the Pension Fund on behalf of the nurses was £2,667 17s. 6d.; and, as an illustration of the substantial benefit of the Fund, it is mentioned that one nurse, having reached the age limit, was due to leave on January 1, 1906, with a pension of £52 per annum. For service with Guy's Hospital Maternity Charity the Institution provided from its own resources one nurse and 31 nurse pupils in midwifery, who all passed their examinations. As to the financial position, its soundness is conclusively attested by the announcement that the year closed with an excess of assets over liabilities of £2,506, as compared with £2,118 carried forward from 1904.

### HOW TO BECOME A NURSE

Many young women of the present day are, for one reason or another, attracted to Nursing as a career, but are at a loss to know how to enter the ranks of this important profession. "How to Become a Nurse: The Nursing Profession, How and Where to Train," by Sir Henry Burdett, K.C.B. (price 2/4 post free), is the title of a most admirable work, which will be found to be a thoroughly reliable guide to this noble calling. It contains a list of the recognised Training Schools throughout the English-speaking world, with particulars respecting Hospital, Infirmary, District, and Military Nursing, and answers very completely all the enquiries of aspirants who would fain believe that they have a vocation for Nursing.

It is well, however, before entering upon a Probationership, to obtain some knowledge of the duties of a Nurse. These are very fully explained in "Nursing: Hints to Probationers on Practical Work," by Mary Annesley Voysey (price 2/3 post free). From this book a very clear insight may be gained into the character of the training it is necessary to undergo, and of the qualities that are absolutely essential to success in this self-sacrificing profession.

Having decided to adopt Nursing as a profession, it would be advisable, whilst waiting for a vacancy in the Institution of your choice, or in the early stages of your probation, to acquire some knowledge of the elements of nursing, bandaging, and the structure and working of the human body. In this direction, "Nursing: its Theory and Practice," by Dr. Percy Lewis (3/6 post free); "A Practical Guide to Bandaging and Dressings," by Dr. Johnson Smith (2/- post free); "Elementary Physiology for Nurses," by Dr. Marshall (2/- post free); "Elementary Anatomy and Surgery for Nurses," by Wm. McAdam Eccles, M.S. (2/6 post free); and "Elements of Anatomy and Physiology," by Dr. W. Bernard Secretan (2/3 post free), will be found most useful, and the information they convey is clear, concise, and accurate.

It is of course impossible to avoid the use of technical terms in such works as these, and it is therefore necessary that a good dictionary should be at hand for constant reference. "The Nurse's Pronouncing Dictionary," by Honnor Morten (2/- post free), has been compiled especially for the use of Nurses, and contains the definition and pronunciation of most of the terms used in Medical and Nursing treatment. Its size also is a strong point in its favour, as it can very easily be carried in the apron pocket.

Another most useful work, and one it would be well to obtain, is "Surgical Instruments and Appliances," by Harold Burrows, F.R.C.S. (1/8 post free). This book is of the utmost value to the Probationer, as by its assistance she will be able to readily identify the instruments and appliances used in various operations.

Many other important works on Nursing and kindred subjects will be found in the catalogue of The Scientific Press, Ltd., who give almost exclusive attention to the production of Nursing Textbooks; and the fact that their publications are in use in the foremost Training Schools is indisputable evidence of their value.

The Scientific Press, Ltd., of 28–29 Southampton Street, Strand, London, W.C., will be pleased to send you, free of cost, on receipt of post-card, a copy of their latest Catalogue of Nursing Manuals, Charts, Case-Books, &c.

### ENGLISH NURSES AND PROSPECTS IN BRUSSELS.

With reference to the statement in a widely circulated weekly journal that there is great scope for English nurses in Belgium, we warned our readers not to rush over to Brussels without definite information. We have since heard from a well-known medical man in the capital that, in his judgment, the establishment of a Nursing Home, started by English nurses, who are not only properly trained and certificated, but are also fluent French scholars and good women of business, is a project that offers reasonable chance of success. A considerable amount of capital would, of course, be required to furnish a home in a good locality to receive patients. Our informant, who, although a Belgian himself, is in touch with English medical men, also thinks that there is an opening for perhaps two English nurses in the capital, but certainly not more at present. Besides sisters of charity, nursing in Brussels is almost entirely in the hands of a lay institution, whose staff, we understand, belongs to the servant class and are not fully trained. As to other parts of Belgium, we may have more to say another week.

### ANOTHER CASE OF INTERFERENCE WITH A MATRON.

Following the case of interference with the duties of the matron at Meath Infirmary, we have received a complaint respecting a similar departure from the sound principle of either placing confidence in high officials, or dispensing with their services, in the North of England. At the instance of the matron of this institution, one of the probationers, whom she had trained and who had turned out a good nurse, was promoted to be charge nurse. But it transpired after a time that she was not at all satisfactory in the administrative capacity of her new position, the matron having repeatedly to find fault with her generally. Each time she resented this, and she also threatened to appeal to the Chairman of the House Committee, with whom she is acquainted in private life. The matron hesitated to report her conduct, having so strongly recommended her to the Guardians; but the nurse, realising that she could not have it all her own way, resigned her position, and reported the matron to the Chairman of the House Committee, asserting that the matron made her life miserable. When the resignation was announced at a meeting of the Guardians, the Chairman of the House Committee said he had heard privately that the nurse was being ill-used and was forced to resign; and, as the result of an altercation, an inquiry was subsequently held in the matron's office. The nurse was there allowed to recite her grievances, which the matron was desired to refute, and in the end the Guardians asked the nurse to withdraw her resignation and "try to live happily with the matron." We should like to know how the Guardians imagine that the discipline of the training school can be maintained by action of this kind. A nurse, of course, has a right to state the reasons for her resignation, but they should be given along with the intimation of her intention, and not reserved for the ear of an individual member of the governing body.

### DINNER TO AN AUSTRALIAN MATRON.

An interesting dinner was given by Miss Glover at the Vienna Café, Melbourne, on March 26, to bid God-speed to Miss Burleigh, matron of the Melbourne Hospital, and Miss Brayshay, Hon. Secretary of the Victorian Nurses' Association, prior to their departure to England for a holiday on the *Afric* and *Marmora* respectively. After the toast of "The King" had been honoured by the company, which consisted entirely of ladies, Miss Glover, in proposing "Our Guests," paid Miss Burleigh and Miss Brayshay a high tribute for the manner in which they had maintained the standard of their profession; and in response to the toast of her own health, the hostess delivered an eloquent speech on nursing matters. During the absence of Miss Brayshay from Melbourne Miss Glover will act as Hon. Secretary of the Victorian Nurses' Association.

### THE WORK OF THE EAST-END MOTHERS' LYING-IN HOME.

At the annual meeting of the East-End Mothers' Lying-in Home, which takes place on Friday afternoon this week at 3 Grosvenor Place, the reports of the Committee of Management, the visiting medical officer and lecturer, and the resident lady superintendent will be read. In the first it is mentioned that the fees earned by the training of pupil midwives and nurses were nearly £100 more than in the previous year, but that, on the other hand, expenses of the Nurses' Home exceeded the receipts by £132. The visiting medical officer, referring in his report to the Home, states that many cases in the out-patient department have caused considerable anxiety and work to the nursing staff, and that some of these were removed to the Home, owing to insanitary surroundings of home life. He expresses the hope that the good work of the Home may be continued without interruption and may meet with hearty support. Miss Margaret Anderson, who only succeeded Miss Alice Blomfield on June 1 as lady superintendent, reports that the Midwifery Training School continues to make satisfactory progress, and refers to the fact that after the last examination the Central Midwives Board approved of the Home as a school for training under the Act. The number of visits paid by the day and night staff last year was 7,587.

### LADY LONDONDERRY ON THE NURSE AND THE MOTHER.

In a speech at the annual meeting of the Comber Nursing Society Lady Londonderry said she thought that one of the most useful works a trained nurse could do was to explain to a mother the irreparable damage she does her child when she will not carry out, if she is able, her first duty to her offspring, and also to urge upon her the moral, as well as the physical, necessity of nursing her child, and thereby developing in herself that supreme instinct of maternal sympathy which is of incalculable moral worth to herself and to her child in coming years. Lady Londonderry strongly insisted that it is "a mother's duty to feed her child for nine months after its birth by a remarkable transmutation of her own life-blood, because if she be in health no other device is of equal value for her child." We agree with her that those who are nursing the mothers back to health after their confinement and giving proper attention to the child for the first fortnight, have valuable opportunities, not to say obligations, offered them of exercising the most salutary influence.

### PAUPER ATTENDANTS AT MIDLAND POOR-LAW INFIRMARIES.

The report of Mr. E. B. Wethered, the general inspector of the Local Government Board for the district which comprises the Union County of Gloucester and parts of the counties of Hereford, Somerset, Stafford, Wilts, and Worcester, shows, we are glad to say, that the number of nurses on night duty is nearly double what it was in 1899. The respective figures are 82 and 43. The number of indoor sick was 3,422, as compared with 3,432 on the same date in the preceding year; the nurses, day and night, in attendance numbering 285, against 274. This is another sign of progress, and so is the reduction of pauper attendants as compared with 1899, when there were 312. But, as compared with the year 1904, the number is not satisfactory. In fact, the increase from 159 on January 1, 1905, to 175 on January 1, 1906, indicates retrogression. So far as nursing by pauper attendants is concerned, there should be a steady decrease of the number until there is a President of the Local Government Board who feels himself strong enough to insist that they shall all be dispensed with.

### NURSING UNDER CHRISTIAN SCIENTIST AUSPICES.

At the inquiry which commenced last week into the death of Major Whyte evidence was given by Miss Minnie Robson, a certificated nurse. Miss Robson stated that Major Whyte came to her home in Westbourne Gardens to be nursed in 1903, when he was being attended by Dr. Huxley and Sir Victor Horsley, and that she subsequently took him to Bournemouth, to a hospital in Grosvenor Gardens, and to Osborne. It was after he left Osborne that she first received from him a letter saying that he was going to try Christian Science treatment. On Friday week she was called to attend Major Whyte professionally again, two days before his death, at 38 Eaton Terrace. Being asked whether there was any evidence of proper antiseptic treatment, she replied, "None whatever, the condition being very offensive." At the resumed inquiry on Tuesday, Miss Edith Jones, of Southfleet, who said she was a trained nurse, but had now become a Christian Scientist, stated that the Major's bedsores were washed with warm water and dressed with lint and bandages which he had brought from Osborne. She declared that while under treatment by Christian Science his condition improved rapidly.

### DIVIDING THE INCOME OF A BEQUEST.

The trustees of the Heath Bequest for Newcastle nurses appear to have agreed upon an excellent distribution of the income, which amounts to nearly £200 a year. Half of the amount is to go to the nurse-training institutions in the city, and the other half to the nurses' homes. Prizes amounting to £52 10s., with a silver medal, are to be awarded annually to the nurses of the Royal Victoria Infirmary; £15, in five prizes, being assigned to the probationers; £20 in four prizes, and a silver medal to the nurses up for their final examination; and £17 10s. in three prizes to the sisters. Prizes to the extent of £10 will be awarded for a competitive examination among the nurses of the Children's Hospital, the Lying-in Hospital, the Infectious Diseases Hospital, and the Union Hospital. The trustees will also hand over one-fourth of the premiums paid by the nurses in the different nurses' homes in Newcastle and Gateshead to the Royal National Pension Fund for Nurses not exceeding a sum of £2 to any one nurse in one year.

### THE MATRONSHIP OF BRECON INFIRMARY.

A short time ago the post of matron of Brecon Infirmary was declared vacant, and in reply to the advertisement a number of applications were sent in. The secretary has just written to the various candidates, returning their application, testimonials, and photograph, announcing that, in consequence of the present matron having withdrawn her resignation, there is no vacancy. Regret is very properly expressed in the letter at the trouble given to the applicants.

### DISGRACEFUL ASSAULT ON A NURSE AT A LONDON INFIRMARY.

At the North London Police Court last week a man of 54, who had been a patient at the Holborn Union Infirmary, was charged with an assault on one of the nursing staff. The nurse herself, in giving evidence, said that the prisoner complained of a poultice which she had made. When she told him that he could speak to the doctor on the question he got out of bed and struck her in the face, knocking off her spectacles. Hitting her a second time, he broke one of her teeth. The magistrate, who affirmed that nothing could be worse than for a patient to strike a nurse who was ministering to his comfort, sentenced the offender to 21 days' imprisonment—a very slight punishment for such outrageous and brutal behaviour.

### INVALID COOKERY EXAMINATION AT WAKEFIELD.

The second annual examination for practical invalid cookery among the nurses of the Clayton Hospital, Wakefield, was held on Thursday last week. Thirteen probationers who had attended the course of lectures by Miss Pressland, matron, competed. After a very close contest between Nurses S. E. Moore and M. E. Thompson for the first prize, the examiners, Mrs. Statter and Miss Edith Wright, awarded the first prize for the seniors to Nurse S. E. Moore; and very highly commended Nurse M. E. Thompson. The second prize to the juniors was awarded to Nurse M. E. Milligan. The prizes are given by the Chairman of the House Committee.

### SHORT ITEMS.

We understand that Queen Charlotte's Hospital sent in 18 pupil midwives at the April examination of the Central Midwives Board and that they all passed.—A drawing-room sale will be held on Thursday, May 17, by permission of Dr. Octavia Lewin, at 25 Wimpole Street, at 2 p.m. to 7 p.m., in aid of the maintenance of the first patients at the Children's Sanitorium, Holt, Norfolk.—A meeting in connection with the Association for Promoting the Training and Supply of Midwives will be held at 24 Park Lane on Thursday, May 17, at 3 p.m., Lord Balfour of Burleigh in the chair.

### The Nursing Outlook.

"From magnanimity, all fears above;
From nobler recompense, above applause,
Which owes to man's short outlook all its charm."

### HOSPITAL HOUSEKEEPING.

QUITE recently certain newspapers have started the cry that the voluntary support of hospitals is falling off and that many institutions are languishing for want of funds. These statements will not bear investigation, for although it may be true that here and there a hospital, for reasons to which experts can precisely point, may find itself at the moment financially weak, the volume of voluntary support to the hospitals of this country has never been anything like so great as it is to-day. Our view is that the actual money given by the public to the hospitals at the present time is ample to meet the necessary expenditure, providing the management and control are adequate. What the hospitals need is to exhibit a spirit of resolute economy, whereby all demands for new buildings and other extravagances shall be closely examined and controlled.

The hospital need of the moment in fact is efficient housekeeping, and the adequate control of expenditure everywhere. Hospital housekeeping, especially in the smaller institutions, is mainly if not entirely under the control of women. Every such housekeeper has it in her power to win for herself a great reputation by the exhibition of knowledge and skill in the housekeeping arrangements. The amount of waste which takes place in households would, if prevented, represent a sum so large as to provide many families with ample means for the enjoyment of an excellent holiday in the country during their vacation. It is not sufficiently understood, although the numbers to be catered for in each household must necessarily affect the average cost, that it is in fact true that a slight increase in the cost of each individual per bed per week speedily overbalances the gain of a decrease in numbers. One hundred and fifty nurses, for instance, boarded at the rate of 6s. per week will cost £260 a year less than 125 boarded at 8s., and this difference per head in the cost is insignificant, compared with many differences which actually exist, when a careful comparison is made between the housekeeping expenditure at various institutions.

There is no space here for us to enter into details in regard to the housekeeping arrangements of the doctors' and nurses' tables. We may, however, observe that in large hospitals, where there are many resident medical officers, these are often boarded at a far higher rate than in smaller institutions where there are but one or two, although the same gentlemen pass directly from the one class of institution to the other, and cannot be supposed to require better food in one place than another. Again, it is the fact that the cost of food for the medical officers in many institutions is out of all proportion greater than that of other members of the house. In that excellent book, which every hospital matron, steward, and housekeeper should possess, "Hospital Expenditure—The Commissariat" published by The Scientific Press, it is shown that when two bills of fare, taken as actual specimens of the board provided in two large London hospitals for doctors and nurses, respectively, are compared, the one works out at from 21s. to 35s. per week and the other at only 8s. per week. Yet most housekeepers would fail to detect any reason on the face of it why the first bill of fare should cost from three to four times as much as the second. The difference is caused by lack of attention to details, and any matron or housekeeper who will take the trouble to go fully into the matter may easily save from 15s. to 20s. per head on the weekly average cost in one of these two departments alone. We merely give this instance to encourage inquiry and investigation on the part of the matrons and hospital housekeepers, and we shall be very glad to answer any questions or to afford them any assistance at any time.

In one hospital where the course here suggested was followed, a zealous secretary and lady superintendent succeeded, by good housekeeping, in reducing the cost of maintenance to the extent of from £1,250 to £1,700 in one year.

The present wastefulness in public institutions is due largely to the want of system, and a thorough training in diets, marketing and housekeeping generally. In the United States all these matters are taught in special courses in connection with the special schools, and it is the common practice for each hospital to have its own dietician, who is responsible for the administration of the kitchen, its economical management and the supply of food generally. There can be no doubt that a similar system would effect great economies in the hospitals of this country. Even as it is, as we have shown by the figures just given, every matron, steward and housekeeper who will take the trouble to obtain a copy of "Hospital Expenditure—The Commissariat" and make a study of these questions, should inevitably add largely to their reputation for efficiency, and may produce economical results which cannot fail to receive a handsome acknowledgment at the hands of the managing committees. The need for improved hospital housekeeping is urgent. The way to promote it, as we have shown, is simple enough in practice. The results which study and inquiry may yield in benefit to the hosptals and the officials directly concerned are so full of promise, as to make it the duty of everybody concerned to set to work and do their best to secure the reforms and improvements so urgently demanded by considerations of economy and good business management.

### Abdominal Surgery.

By Harold Burrows, M.B., F.R.C.S., Assistant Surgeon to the Seamen's Hospital, Greenwich, and to the Bolingbroke Hospital, Wandsworth Common.

### AFFECTIONS OF THE STOMACH.

### Complications of Gastric Ulcer.

(*Continued from p.* 62.)

*Pyloric Stenosis and Dilatation of the Stomach.* In cases of chronic ulceration a large amount of scar tissue is produced in the immediate neighbourhood of the ulcer. And scar tissue in the stomach, like scar tissue elsewhere, undergoes gradual contraction. So that a long standing gastric ulcer, whether innocent or malignant, may narrow the circumference of the stomach to such a degree that there is obstruction to the passage of food.

Most often such narrowing, or stenosis, occurs in the pyloric region, but occasionally it is situated near the middle of the organ, and in this case it produces what is described as "hour-glass" contraction of the stomach. The symptoms are much the same in the two conditions, and the description of pyloric stenosis given below may be applied with slight modifications to hour-glass stomach.

As a rule, the patient gives a history of indigestion lasting through some years, and perhaps he has in the past suffered from hæmatemesis or severe pain and vomiting after meals—symptoms which are strong evidence of gastric ulceration. In any case his present complaint probably includes vomiting, a feeling of discomfort in the epigastrium, constipation, feeble appetite, lassitude, and loss of weight. Of these the first named is the most characteristic, for it is unlike vomiting from most other causes. It is intermittent, occurring perhaps once in 24 or 48 hours, and frequently is not accompanied by severe nausea, retching, or pain. The quantity ejected on each occasion is large, and the vomited matter is sour smelling.

The most marked anatomical result of pyloric obstruction is dilatation of the stomach, which may become so large as to reach to the lower part of the abdomen.

On inspection of the abdomen in advanced cases, and especially in thin and wasted patients, peristaltic waves may be distinctly seen passing along the distended stomach. This visible peristalsis of the stomach is characteristic of pyloric obstruction. To estimate the amount of dilatation of the stomach the organ is inflated, as described in a previous article.

The treatment of dilatation of the stomach due to pyloric stenosis may be palliative or operative.

Nearly all cases will be benefited to some extent by regularly washing out the stomach every day, by careful dieting, and by suitable medicinal treatment; and even if an operation be deemed advisable, such measures ought to be employed beforehand, because with their aid the patient may be rendered in a better condition to bear the operation. The best time for washing out the stomach is just before the patient retires to rest; but whatever hour be selected, it must allow time for the patient to digest his previous meal, for which purpose an interval of three hours is sufficient. This somewhat obvious necessity is occasionally overlooked, with the result that a patient is deprived regularly of his supper soon after he has taken it, and instead of being benefited by the treatment he gets thinner and weaker every day from starvation.

Although when properly employed gastric lavage combined with other measures is of considerable value in the treatment of pyloric stenosis, it does not cure the patient; and, except in rare instances, the patient will not be able to discontinue the use of the stomach tube without a recrudescence of his former troubles. Moreover, the stricture of the pylorus is apt with time to become more and more pronounced. For these reasons an operation is almost sure to be required sooner or later, and as a rule the earlier it is performed after unmistakable symptoms have arisen the more satisfactory will it be.

### Gastro-Enterostomy.

The operation employed in the majority of these cases is gastro-enterostomy.

The object of the operation is to make an artificial opening between the stomach and the small intestine, so that food which is unable to find an exit through the pylorus may escape through the artificial opening.

In preparing a gastro-enterostomy the details are similar to those required for other abdominal operations. In addition, the surgeon may wish to have the patient's stomach washed out by means of a tube immediately before the operation commences. Another point that needs special emphasis is the necessity for keeping the patient's mouth as celan as possible; and it is a common custom to order the use of an antiseptic mouth-wash or a gargle just before the patient comes to the operation room.

### Tetany.

There is a curious complication of gastric dilatation which is worth mentioning, and this is tetany. It is especially apt to occur in long-standing cases, and the actual seizure not infrequently ensues immediately on the introduction of a tube for the purpose of washing out the patient's stomach. In other cases it follows close upon a severe attack of vomiting.

A seizure of tetany commences with numbness and tingling of the hands and feet, especially of the hands. The attack may not progress further than this. In severer cases there soon follows, it may be almost immediately, a painful spasmodic condition of the muscles. The arms are flexed and rigid, with the thumb adducted towards the little finger. The knees are drawn up, and the patient is unable to extend his legs. The pain caused by these spasmodic attacks is severe.

The occurrence of tetany is a strong indication for operation in cases of dilatation of the stomach; otherwise the tetanic seizures are nearly sure to be repeated, and the patient will most probably survive but a short while. It is hardly possible to overlook a severe attack of tetany, or to mistake it for any other condition; but it is easy, unless special care be taken, to fail to notice its minor manifestations, such as numbness and tingling in the hands and feet, cramps in the legs and arms, and slight attacks of giddiness; and the nurse should be vigilant to observe and report to the surgeon any complaint of this nature that may be made by a patient suffering from a gastric disorder.

### Congenital Hypertrophic Stenosis of the Pylorus.

There are other causes of dilatation of the stomach than gastric ulcer, and these may be briefly mentioned. They are gastric adhesions, especially those due to inflammation of the gall-bladder, duodenal ulcer, malignant disease of the stomach, chronic gastritis, and congenital hypertrophic stenosis of the pylorus. Congenital hypertrophic stenosis of the pylorus is a disease of early infancy. A baby, who was quite well at birth, begins to vomit when it is a few days or weeks old. The vomiting is usually in the course of a meal or shortly afterwards. Gradually the vomiting increases in quantity and in frequency, and the infant loses weight.

The treatment is to change the diet and to wash out the stomach by means of a tube once or twice a day. If vomiting continues and the child still loses weight, an operation must be done without delay. The great indicator of the success or failure of treatment is the infant's weight, and this should be taken and recorded every day.

### Carcinoma of the Stomach.

Malignant disease of the stomach may cause symptoms indistinguishable from those of simple gastric ulcer; and in these cases the differential diagnosis can only be made by analysis of the gastric contents removed after a test meal. In other cases, in addition to symptoms of gastric ulcer, such as indigestion, dilatation of the stomach, etc., there are well marked anæmia, rapid loss of strength and weight, and loss of appetite. In yet other instances the anæmia and loss of flesh are the only indications of the disorder. The treatment in certain cases, especially those which are in an early stage, is excision of the malignant growth. But usually by the time the disease is recognised it has advanced too far to be cured by operation, and only palliative measures are available. Those are, firstly, washing out the stomach regularly the last thing at night, together with careful regulation of the diet, and suitable medicinal treatment; secondly, gastro-enterostomy. In many cases which are too far advanced for a radical operation, the patient may be greatly relieved, and the short remainder of his life made comparatively comfortable by the regular use of the stomach tube, combined with proper dietetic and medicinal treatment. With these and the care of a good nurse the patient may go to the end of his journey quietly and peacefully, unharrassed by the troubles that otherwise are inseparable from cancer of the stomach.

### The Nurses' Clinic.

### THE DISTRICT NURSE AND ECZEMA.

With the exception of eczema all forms of skin disease are rare in district work, but eczema of all kinds and in all degrees is extremely common in chronic cases. Complete recovery is unusual; a small patch is generally left that defies all treatment, and after a lapse of time, varying with local conditions and constitution, the patch spreads; and there are many cases where, although the disease varies in intensity from the barely perceptible to the almost intolerable, it is practically never absent. Nevertheless, careful treatment invariably results in great alleviation, and therefore every case is hopeful.

A year or two ago I was sent for to nurse a severe and previously neglected case of eczema. The patient was a middle-aged woman who had no one to do anything for her, but a husband out at work all day and a half-witted daughter of sixteen just able to do rough cleaning under constant direction. Hands, arms, feet, legs, and abdomen were covered with scabs and discharge, but head and face were entirely free. Eczema is said to be non-contagious, but in district work I always treat it with the same precautions as forms of skin disease acknowledged to be infectious, burning scabs and old dressings, soaking rags intended to be washed for further use in carbolic, and so forth.

The first thing to be done in all cases of this kind is to remove the scabs, as until that is done the lotion ordered by the doctor cannot be applied. There are various ways of doing this; the diseased part can be bathed with warm bran-water, oatmeal water, or milk and water until the scabs are loosened and can be cautiously picked off with forceps. This is a tedious business, especially if the area affected is large, and the work is best done by applying a poultice, leaving it on all night, and thoroughly bathing the surface with warm oatmeal water the next morning, repeating the process from the beginning if all the scabs are not loosened. Warm linseed-meal poultices are often used, but probably the most soothing, cleanly, and effective poultice is made of boiled starch spread half an inch thick on white calico, and allowed to become icy cold before being applied. As soon as all the scabs are removed the lotion ordered, and later on the ointment, can be used.

In all serious cases (except perhaps of eczema affecting the scalp) the patient should be advised to remain in bed. All extremes of temperature must be avoided; wine, spirits, etc., must on no account be taken; the diet must be light, digestible, and non-stimulating. Baths of starch water or oatmeal water may be found soothing. The patient must be warned to resist the inclination to rub or scratch the skin, and in the case of young children, measures must be taken to prevent them from doing so, by protecting the affected part and fastening the arms loosely to the side. If the irritation is intense it may cause sleeplessness, inability to take food, and consequent exhaustion. When this is so the doctor must be informed at once, and he will order suitable sleeping draughts or other treatment. The patient referred to recovered in about three weeks.

Eczema in infants seems generally to arise from dirt; at any rate, it almost invariably yields to cleanliness and care. The most common form is eczema of the scalp. It usually starts behind both ears, and the entire scalp, possibly the face in addition, soon becomes a mass of irritating sores. The first treatment is to soak three layers of lint or of soft old linen in warm olive oil and cover the scalp with them; green protective tissue should be laid over the lint, and the tissue well covered with cotton-wool, and the whole held in

### THE NURSES' CLINIC—*Continued.*

place by a cap formed of a triangular bandage. If the face also is sore, a three-fold mask of lint should be soaked in oil, covered with cotton-wool, and held in place by strings, two passing above the ears and tied at the back, and two below. The next day the head must be washed with warm oatmeal water, and as much of the hair as possible cut away; a repetition of the treatment will probably be required. When all the scabs are gone calamine lotion is generally applied.

In some cases infants are affected from head to foot; baths of starch water or bran water may be soothing, and then the child must be swathed in oiled rags until the skin is clear enough to apply calamine lotion. Mothers must be specially warned to keep a separate towel for the child, and always to wash it in a separate basin of water, as no vestige of soap must touch its skin. Cardboard splints, well padded, may be needed for the child's limbs.

In cases of chronic eczema the sufferer should be warned to give up the use of soap on all parts that are or have been affected, and to keep the skin as dry as possible—a difficult matter if the patient is a woman and if, as so often happens, the eczema is on her hands. A good dusting powder is made of one part starch, one part zinc, and two parts boracic.

In the mild but very persistent cases of eczema of the scalp found among otherwise healthy young people, a little boracic ointment rubbed in with the tips of the fingers will be found soothing. Medical advice should always be obtained.

Eczema is caused in certain persons by some special irritant, and if this can be discovered and avoided the disease will not recur. In some instances the handling of such generally harmless articles as sugar and flour will occasion it, and a change of occupation is sometimes found necessary before it can be cured.

### Incidents in a Nurse's Life.

### DADDY'S WILL.

Some patients who pass through the hospital wards are soon forgotten; others for some reason leave a lasting impression on the memory. One patient, whom I still remember well, was an old man of eighty. He was not by any means a handsome old fellow, but his snow-white locks gave him a venerable appearance, and in his youth he must have been a "well-set-up" man. Daddy, as we called him, came in for heart trouble with complications. At first he was in bed, but after a week or so the doctor gave him leave to get up, and later on he used to stump out into the garden, where he would sit under the trees, smoking his pipe, gossiping with an old crony or two, and generally enjoying himself. Poor old man; his pleasure was of short duration, for before long he was confined to the ward, then to bed, and soon was not even able to bear our getting him, rolled up in blankets, into a chair by his bedside.

Now Daddy was a "gentleman of means," so when his rapid failing became evident to his relatives we began to hear a great deal about "the will," he being a tailor by trade, and bearing the reputation of charging a "tidy sum" for a pair of trousers or a coat. Sister, in particular, was interested, as the old man consulted her on the all-important subject, and gave her much of his confidence. It appeared that Daddy had two sons, the one lived in "Lunnon," the other at home near his father, and on the strength of "nearest ought to be dearest" expected to get all the old man's money. He was a little fat man, round like a tub, with cheeks of a ruddy hue. Bob, as he was called, came to see his father very often in those days, and we used to feel very sorry for the son in "Lunnon" whom Bob was trying to do out of his share of the money, especially as the old man had told us he had been a good son, and really seemed to have a soft corner in his heart for him.

At last the climax to matters came. One evening Bob informed Sister, in rather a sheepish way, that he and another man, a supposed lawyer, were bringing Daddy's will that he might sign it that night. Sister made no remark, but decided privately to be present at the signing of that will, feeling sure that they were going to treat the old man unfairly. However, in the rush of a busy time Sister forgot her intention, and it was only by accident that she went into Daddy's ward. He was sitting propped up in bed, a bright spot of colour in each cheek, and the pen held in his poor trembling old hand, whilst the men instructed him as to the place to sign. It was at this critical moment that Sister entered, and with a quick glance she took in the situatión, and before his trembling hand could make a stroke she was at the old man's side. "Daddy," she said, "have you read the paper? Do you quite understand what you are about to agree to?" "No," he answered, "but they have told me what is in it." Sister's reply was to take the paper, and in a clear voice to read the contents, in spite of the opposition and the disappointed looks of the men. Great indeed was the poor old man's horror to learn that but for Sister's intervention he would have left all his money to Bob, and none to the loved Benjamin.

Daddy only lived a short time after this, and a few days before he died he looked up into Sister's face. "My dear good woman," he said, "I almost wish I had left all my money to you."

### A Visit to the Midland and Birmingham Women's Hospital.

It is now upwards of six months since the staff of the Midland and Birmingham Women's Hospital commenced work in the new building, over which the lady superintendent, Miss Richmond, kindly conducted me the other day. If in the olden days some primitive methods had to be endured, together with the feeling—as Miss Richmond whimsically expressed it—"that the hospital got hopelessly older and older and older"—the staff now appears to enojy every modern improvement.

The hospital, a pretty red-brick building, standing just away from the main road yet conveniently near a busy, poor, but healthy part of the city, is built in two blocks, one containing the nurses' quarters, offices, dispensary, kitchens, etc., the other the wards and two theatres. The blocks are connected in the centre by a long corridor with doors opening on either side to the garden. There are four large free wards and four small private ones, comprising accommodation for fifty patients altogether. The prevailing atmosphere on entering is a snowy freshness, thrown out by the soft whiteness of walls, doors, and counterpanes. The warm brown of polished floor and varying shade of spring flowers give colour, while a happy group of patients seated in comfortable basket chairs round the fire, baby worshiping, their object, a jolly little babe, three weeks old, the result of a Cæsarian section, provide a human element. Elegant balconies are attached to the wards, approached through glass doors, and overlooking the garden. In the theatre were two nurses busily "cleaning up," and one kindly posed for a snapshot to be taken in the gown and mask worn whilst assisting at operations. The mask was first introduced in England by one of the hospital's surgeons, who had seen it adopted in Germany.

The lady superintendent arranges for as many nurses as possible, besides all who are off duty and care about it, to see the special operations, so that the nurses have the advantage of becoming familiar with those of a nature which in general work are more rare. Having seen the wards, theatres, dispensary, steam laundry, and mortuary-chapel (a peaceful little room, more chapel than mortuary, bearing every evidence of innate and far-seeing thoughtfulness) we went to the nurses' quarters.

Each nurse has a bedroom to herself, suitably furnished in oak; the use of the reading and writing room, containing a well-filled bookcase, a comfortable dining-room, and a delightful recreation-room. The latter looked really charming, with a crackling fire throwing out cheery rays, lighting up and harmonising with soft tones of carpet and

Ready for Operations.

hangings; numerous chairs, sofas, and cushions inviting repose and restoration of jaded nerves and from the prettiest of windows the promise of spring awakening. The garden is plentifully planted with trees and blest with a full-sized lawn, adaptable for croquet or tennis, as the nurses ordain. Near a winding path I descried a number of little plots—the nurses' gardens—all laid out with professional neatness, but not yet revealing the gardener's secrets. Overlooking the garden, on the other side of the road, the convalescent home stands, fashioned and arranged after the manner of a country homestead. We surprised a merry party of convalescents at tea, and remarking on their excellent spirits the lady superintendent pointed out that one advantage of having the home so near was that the patients could be removed at an earlier date than usual from the invalid atmosphere of the wards and thus "picked up" much more quickly.

The nursing staff consists of three day, one night sister, and nineteen nurses and probationers. The period of training is three years, but suitable candidates are received at the age of twenty or twenty-one, so that the training can be completed by the time they are old enough to enter a general hospital. Two weeks' holiday is given the first year, three the second and third; two and a half hours off duty daily and half a day every fortnight; on Sunday half the day twice a month and church time other Sundays. The salary is £8 the first year, £12 the second, £16 the third, and £25 to staff nurses. Except in fairy tales all good things come to an end, so, after a pleasant chat in the matron's sitting-room, I found myself on the main road once more, thinking somewhat wistfully of the glamour and possibilities of early training days.

### Central Midwives Board.

### CHARGES UNDER THE ACT.

A meeting of the Board was held on Thursday last for the consideration of charges brought against certified midwives. There were present Dr. Champneys (Chairman), Mr. Fordham, Mrs. Latter, Miss R. Paget, Miss Wilson, and Mr. Parker Young.

The first cases heard were those of Mary Alice Jackson and Elizabeth Pattillo, certified midwives, against whom Dr. Mary Smith, Inspector of Midwives in Manchester, appeared.

The charges against Mary Alice Jackson were that she was intoxicated while on duty in attendance on a patient, and that she disobeyed rules E 1, E 2, E 3, and E 7 on the same occasion. Dr. Smith stated that she had been suspended for not carrying appliances and wearing a washable dress, but was found to be still practising. Dr. Smith found her intoxicated and incapable and without appliances and antiseptics: the doctor conducted her home and called in another midwife to the case. The woman's explanation was that she was suffering from "nervous exhaustion." The Board decided to remove her name from the roll.

In the case of Elizabeth Pattillo the charges were that she failed in a certain case to apply proper and sufficient ligatures to the umbilical cord, whereby hæmorrhage ensued, causing the death of the new-born infant; that she failed to notify the local supervising authority of the child's death, and that she did not keep a register of cases. The coroner's verdict at the inquest on the infant had been that death was due to hæmorrhage at birth owing to the ignorance of the midwife. It appeared that the hæmorrhage was not noticed until three hours after birth; the woman's statement was that she tied the cord twice with tape and that when she left the child half an hour afterwards, having washed it meanwhile, everything was perfectly all right, and she supposed the tape must have slipped. As to notifying the local supervising authority, she had sent word of the death to the police-station and thought that was sufficient. Dr. Smith said that the woman was rather deaf and not intelligent, and that though she repeatedly had gone over the rules with her, she seemed very slow at grasping them, and did not keep her register completely at all. The Board decided to severely censure the woman, and requested Dr. Smith to inspect and report to them again in three months' time. They felt that it was not proved that it was through neglect on the part of the midwife that the infant had died; the other two charges they considered proved.

The cases of Sarah Hine and Mary Ann Wallis were heard together, as the charges were identical. They were both accused of taking no appliances or antiseptics with them when called to a confinement and of not keeping or possessing a register of cases and declining to provide themselves with one. Dr. Meredith Richards, of Croydon, appeared against them. They both expressed a desire to be removed from the roll as they had no intention of complying with the rules; Sarah Hine, however, refused to return her certificate unless the fee of 10s. were refunded to her. The Board decided to remove their names from the roll, and the Secretary was requested to inform Sarah Hine that if she used her certificate in any way she was liable to a fine not exceeding £5, and that her safest course was to return it without delay.

The case of Mary Ann Stead, against whom Dr. Worthington, of Lowestoft, and Miss Swain, Inspector of Midwives for the County of Suffolk, appeared, was then heard. The charges against her were that, being in attendance on a patient, she had disobeyed rules E 2, E 3, E 7, and E 11, and that the patient being ill and her progress unsatisfactory, she did not decline to attend alone or advise that a registered medical practitioner be sent for. From statements made by the midwife to Dr. Worthington, who was called in on the seventh day after the confinement (October 30), the woman was going on well until November 3, when she had some sort of fit; on November 5 she had two fits and another on November 6, just before Dr. Worthington arrived about 1.30 A.M. Dr. Worthington said it was evident that the patient had had rigors; she was suffering from puerperal fever, was sent to the hospital, and there died on November 11. He was certainly of opinion that he should have been sent for sooner; the husband, who was a late groom of his, had come round for him; and he had received no written message. Miss Swain, who saw the midwife on December 5, stated that she did not make any mention of fits to her, but said that the patient was of a yellow colour on November 3, though her temperature was all right. Miss Swain found, however, that Mrs. Stead did not really understand the taking of a temperature. On the night of the 5th she (the midwife) had told the husband he must send for a doctor. The midwife admitted that she used no antiseptics and only washed her hands before leaving home, and said that the patient objected to being washed again after she had washed her twice. The Board decided to remove her name from the roll.

Miss Swain also appeared against Harriet Fields. The charges against her were that being in attendance on a patient she disobeyed Rules E 2, E 7, E 11, and E 19, and that the patient being ill and her progress unsatisfactory on the fifth day she did not, until the seventh day, decline to attend alone or advise that a registered medical practitioner should be sent for. Miss Swain showed that she had repeatedly told her that she must get antiseptics and appliances, but she took no heed, that she was not a clean woman, and that she admitted the charges in this particular case, where the woman died of peritonitis. The Board decided to remove her name from the roll as she had deliberately failed to provide herself with appliances, and a woman had died apparently through her neglect.

Two charges of intoxication were brought against Emma Jones, certified midwife. Miss Mackenzie, Health Visitor and Inspector of Midwives for the Warwickshire County Council, stated that she had seen her drunk in her own home. The husband of one of the patients appeared and gave full details of the midwife's helplessly drunk condition. A friend, who had been called in by the husband, also gave evidence to the same effect. In the second case, the child died, and in giving the verdict at the inquest, the coroner had referred to the midwife's "chronic drunkenness." In this case she was found drunk in the garden with the infant's corpse in a box beside her, having undertaken to convey it to the churchyard. The Board decided to remove her name from the roll.

The last case heard was that of Emma Whittaker. The charges were that she had disobeyed rules E 1 and E 2, and that being in attendance on a patient, she had omitted to notice that a portion of the membranes belonging to the afterbirth had not been expelled, and that she had not taken the patient's temperature nor sent for a doctor when her progress was unsatisfactory. The coroner's verdict had been that the patient died from septic peritonitis, due to the neglect of the midwife. The Board decided to remove her name from the roll.

At the conclusion of this meeting a special meeting of the Board was held to consider the question of new offices. It was decided to give powers to the sub-committee to lease new offices at a rent not exceeding £400 per annum, including all charges, for a period of seven years. A letter from the clerk of the Privy Council in respect of the revised rules was considered. The letter referred, among other details, to rule F 2 (new rules), wherein the Board claimed power of suspension of a midwife. The Privy Council had been advised that no power of suspension is conferred upon the Board, the only mention of suspension in the Act being in Section 8 (3), which conferred that power upon local supervising authorities, and they therefore felt Rule F 2 must be omitted.

### Rural Midwives' Association.

### ANNUAL MEETING.

There was a good attendance at the third annual meeting of the Rural Midwives' Association, which was held at 3 Grosvenor Place on Thursday last week.

The chair was taken by Sir Michael Foster, K.C.B., who in his opening speech remarked that the real object of the Association was to promote the welfare of the mothers of the working classes, and to ensure their suffering as little inconvenience as possible from the working of the Midwives Act. He thought that the Association was to be congratulated on the progress made during the past year; the number of applicants for training had largely increased, and the number of demands for midwives had also increased. It was a notable fact that of all those who had gone in for training only ten had fallen away and taken to other occupations. The Association had received most encouraging letters from various parts of the country, showing the good work that was being done. The finances, however, were not in so excellent a condition as the work. They were not as yet in debt; in fact, they had a small balance in hand, but this state of things had only been brought about by the adventitious aid of jumble sales. The Association ought to depend for its income entirely upon its annual subscriptions, and jumble sales, etc., should only be reckoned as luxuries.

The annual report having been read by Mrs. Heywood Johnstone, Chairman of the Executive Committee, Sir Michael Foster moved, and Dr. Boxall seconded, its adoption. Lady Norah Hodgson then distributed a number of nurses' good service certificates, in place of Lady Esther Smith, who was unfortunately prevented by illness from being present. Only one nurse attended in person to receive her certificate, the others being taken charge of by ladies belonging to the various district Associations.

Mrs. Heywood Johnstone then addressed the meeting on the training and inspection of midwives and the registration of nurses. With regard to the training, she said that it was necessary that the women should be very thoroughly taught the theory and practice required for their vocation, for on them depended the life of the mother and child, as, with all safeguards, it often happened in rural districts that the midwife was left alone at such times. On the other hand, it would be a national injustice to bar the employment of thousands of these women of the working classes by too high a standard of examination. Looking at the records of the various nursing groups where midwives and maternity nurses were employed, they found that the results were most satisfactory, many nursing associations having no fatalities at all. As to the inspection of midwives, this had been splendidly organised and carried out by the county Medical Officer, but in her opinion the local Medical Officer of Health was not the right person to act as inspector, as in many cases he had nothing to do with obstetrics, and his duties as sanitary inspector made it inadvisable for him to come in contact with the midwives' equipment, etc. Touching upon the Bill for the State Registration of Nurses, which would make all nurses who had not served three years in a hospital unregistered, and therefore to the ignorant mind undesirable, if not impostors, she said, it had been sugested that women with a short training, who had worked in a district for three years under supervision, should have the same privileges as hospital nurses.

Dr. V. E. M. Bennett, speaking of the old style of midwives, said that they often possessed a vast amount of clinical experience, were better able to stand night work than the younger women, and if treated tactfully they showed themselves very glad of teaching and help. When one of these women proved both dirty and drunken it was better to get rid of her; when she was only dirty she might be reformed. The Education Department of the London County Council had arranged courses of lectures to midwives at various centres, which had been well attended and much appreciated. In smaller places it would be a good plan if the local inspector had a day at home, when these women might come to have their difficulties explained.

During the discussion which followed, in which Mr. Allan Hodgson, Dr. Handford, and Dr. Bostock Hill took part, the statement that the Medical Officer of Health had to perform the duties of a sanitary inspector was traversed, and Mrs. Heywood Johnstone subsequently explained that she had in her mind the Medical Officer of Health for a small rural district in Surrey.

### The Training and Registration of Asylum Nurses.

At the meeting of the Northern and Midland Branch of the Medico-Psychological Association last month Dr. Harding opened a discussion on the training and registration of the asylum nurse. After stating what he considered ought to be the minimum training of an asylum nurse, he mentioned that a three-years' course of training had been in existence at Berry Wood for sixteen years, but that only about 16 per cent. of the nurses who entered the service gained the certificate. He spoke in strong terms of the inadequacy of the system of training and examination of nurses carried on by the Medico-Psychological Association. He pointed out that the only thing in which there was uniformity was the written examination—the least important method of gauging a nurse's efficiency. There was, however, no uniformity in the more essential parts of the examination, while the candidate might actually have had no practical sick-room training at all. He affirmed that the ignorance of some holders of the certificate proved this fact. The value of the certificate was therefore indefinite, and conveyed no indication of the knowledge and experience of the holder, who might be well equipped for her work or who might be the reverse. Thus a great injustice was done both to the able and well-trained women holding the certificate, and to the public, who had no satisfactory guide as to the capacity of the nurse they might employ. It was therefore absurd to claim that the restriction of the right to register to the holders of the Medico-Psychological certificate was any guarantee of efficiency, but rather the reverse. While recognising and giving full credit to the Association for their past work with regard to nursing, he urged the unfitness of a self-elected body of medical men to act as the sole arbiters to decide what the training and examination of a mental nurse should be. It was absurd that no one, however able and interested, should have anything to say on the question of the nursing of the insane unless he or she were a member of the Association. Its Education Committee contained no representative either of the public, of general nursing, or of the mental nurses themselves. He referred to the Report of the Select Committee of the House of Commons on the registration of nurses, and pointed out the different treatment allotted to hospital nurses as compared with asylum nurses. He advocated the representation of asylum matrons and nurses on the central body, and held that this body should exercise the same supervision over the training of nurses in asylums as it will do in hospitals. He also argued that it would not tend to progress in nursing if the qualification for registration in nursing mental cases were restricted to the Medico-Psychological certificate. He supported the view that the qualification for registration should not be restricted to any one certificate, but should be such as might be approved by the central body, thus approximating asylum nursing training to that of hospitals.

### Everybody's Opinion.

[Correspondence on all subjects is invited, but we cannot in any way be responsible for the opinions expressed by our correspondents. No communication can be entertained if the name and address of the correspondent are not given as a guarantee of good faith, but not necessarily for publication. All correspondents should write on one side of the paper only.]

### OUR PRIVATE SALE AND EXCHANGE DEPARTMENT.

"M. B.," who has taken advantage of our Private Sale and Exchange Department, writes : I must take this opportunity of thanking you so warmly for being the medium by which I have been able to dispose of an article which just now is of no use to me, and I feel that the nurse-purchaser will benefit by her little bargain. Your system is so easy, so private, and also very moderate in fee, that it is sure to be widely appreciated.

### POOR-LAW NURSES AND SUPERANNUATION.

"W. M. E." writes : As a nurse I contracted out of the Superannuation Act, 1896. As a matron I now wish to take advantage of the provisions of that Act, but I understand I cannot do so, but that had I as a nurse contracted out of the Act of 1897 I could as a matron have come within the benefits of the superannuation provision. The note in your issue of April 28 conveys to my mind the impression that I can now enjoy the benefits of the Act owing to having become a matron. Is this so, or does the contracting out of the earlier Act deprive me of the benefits aforesaid?

W. Thurnall, Clerk to the Lambeth Guardians, writes from Brook Street, Kennington Road, S.E. : I have read with much interest the article in your issue of April 28, more especially that portion in which it is stated that "the Local Government Board have now determined that if a nurse has contracted out of the Act of 1896 she is bound by her decision in every subsequent appointment she may hold as nurse within the meaning of the Act of 1897; but that if she be appointed to any other office than that of nurse she will come under the operation of the Act of 1896. Thus a nurse on becoming a matron will be allowed to come under the Superannuation Act, . . . and she will be eligible for a pension at the end of her service." I should be much obliged if you could furnish me with the definite authority for the above statement; if it be contained in a letter, the date of the letter and to whom sent. In regard to this subject I should like to observe : (1) That the Local Government Board have repeatedly ruled that notice under Section 15 having once been given by an officer, continues to have effect as regards all new appointments, and cannot be withdrawn. They have also stated that the last paragraph of their circular of February 10, 1898, does not apply in such cases. (2) To rule now that a nurse if appointed to any other office will come under the operation of the Act of 1896 is apparently in direct contradiction to previous decisions. (3) In the event of a nurse being appointed to another office, such as matron, does she of necessity come under the Act of 1896? The statement in the article is that she will come under its operation, but it is subsequently stated that she will be allowed to come under the Superannuation Act. (4) With regard to the statement that "no part of her service as a nurse will be reckoned as part of the service which qualifies for her pension," this is apparently correct. It is true that Section 4 of the Act of 1896 provides that "all service . . . shall be aggregated and reckoned for the purposes of this Act," but the Amendment Act of 1897 provides that nurses contracting out of the Act of 1896 shall not be entitled to receive any superannuation allowance under the said Act. (5) There is apparently no authority under the Act of 1897 to enable an officer who has contracted out to subsequently contract in. I have abstained from making any criticism on the proposals contained in your able article, being desirous of ascertaining in the first instance the exact position in which nurses stand in regard to the Poor-law Officers' Superannuation Acts, and in view of the importance of the subject to these officers, I trust that you will favour me with the information desired.

[*** Our article was based upon a statement published in March that the Local Government Board would, as at present advised, in case of an appeal being made to them under Section 18 of the Poor Law Officers' Superannuation Act, 1896, decide as stated in our article in the *Nursing Mirror* of April 28, page 57. The effect of this decision of the Local Government Board is, we understand, that a nurse who has contracted herself out of the Superannuation Act of 1896 is bound by her decision as regards every subsequent appointment she may hold as a nurse within the meaning of the Act of 1897. But where a nurse has been appointed to any office other than that of nurse as defined in the Act, she then comes within the operation of the Act of 1896. All service rendered as "nurse" will not count for superannuation in such circumstances.—ED. THE HOSPITAL.]

### THE WEARING OF UNIFORM.

"E." writes : I should be much obliged if you would give me your opinion of my wearing uniform, working as I do at a large "medical mission" amongst the slums. I have done so for about ten years, having learnt a great deal not only by lectures, but practically, from the doctor on dispensing, surgery, and district nursing, but I am not a qualified nurse; my work has never been paid for, and I should be sorry to ever take a penny under such circumstances, as I sympathise greatly with the fully trained nurses of to-day.

[We think that, considering the work you are doing and the neighbourhood in which your labours are being carried on, you are entitled to the protection of a nurse's uniform.—ED. THE HOSPITAL.]

"M. D." writes : Much of the correspondence which has been appearing in your columns on this matter of outdoor uniform for nurses is really amusing reading, and I think that the true nurse has reason to exclaim "save me from my friends." Perhaps the wildest idea of all is that the legislature of this country should be asked to regulate the dress of human beings, for this is what an "Experienced Nurse" suggests should be done in your issue of last week. Now, Sir, very few of your correspondents seem to grasp this one fact, that if all nurses in connection with institutions or homes of whatever kind were debarred from wearing their uniform when off duty there would be an end of the trouble. To label a nurse with so many letters in a piece of tin, bronze, or lead, or even silver if you like, would be but an additional target to hit at. It is simply preposterous to suggest that private individuals are not at liberty to dress their nursemaids, parlourmaids, or hallmaids as they think fit, and if you would only raise your voice in support of the plea that nurses shall wear **uniform on duty** only, you would be doing the nursing world of this country one of the greatest services your valuable paper has yet done. I hope that this important question will not escape the attention of those who are guiding the proposed registration of nurses.

### NURSES' EXAMINATIONS AND METHODS OF MARKING.

S. EDGAR MARTIN, M.B., B.Ch., writes from Cleveland Street, Asylum, W. : Having had experience in the Scotch and Irish methods of examining nurses, I am surprised at the system obtaining in certain institutions in England. It appears that the relative positions of examinees as issued by the outside examiner—which positions are not made known to the nurses—are altered by the matron on the grounds of general conduct. I do not take exception to the principle of this system, otherwise than that I am unable to understand why, as in Scotch and Irish institutions, the marks awarded by the examiner and those given by the matron should not be separately stated and published. This method, to my mind, has the following grave faults : It induces the examinees to think that, however high their standard of efficiency in the actual examination may be, they may still, without any explanation, be placed low in the list. Again, it tends to make nurses content with almost any result in the official examination so long as the matron's marks are sufficient to pass them. Of course the question of favouritism can be dismissed without consideration. I should be glad to hear from your readers if this system of not stating marks is universal in English training institutions.

### Appointments.

[No charge is made for announcements under this head, and we are always glad to receive and publish appointments. The information, to insure accuracy, should be sent from the nurses themselves, and we cannot undertake to correct official announcements which may happen to be inaccurate. It is essential that in all cases the school of training should be given.]

ACCIDENT HOSPITAL, MANSFIELD.—Miss Ethelwyn M. E. Wood has been appointed head staff nurse. She was trained at the Beckett Hospital, Barnsley, and has since been staff nurse at the Hospital and Dispensary, Retford. She has also done private nursing at Wakefield.

FEVER HOSPITAL, YOKER, NEAR GLASGOW.—Miss E. M. Marriott has been appointed charge nurse. She was trained at the fever hospitals under the Metropolitan Asylums Board, and has since been staff nurse of the Luton Hospital, Bedfordshire; charge nurse at the Smith Hospital, Henley-on-Thames; and charge nurse at the Fever Hospital at Kilwinning, Ayrshire, Scotland.

HIGH WOOD SCHOOL, BRENTWOOD, ESSEX.—Miss Hannah Mary O'Connor has been appointed assistant matron. She was trained at the Royal Portsmouth Hospital, and has since been ward sister at the Essex and Colchester Hospital, and sister, night superintendent, and acting matron in the Army Nursing Service. In the latter position she was for six months at the Military Hospital, Portsmouth.

HITCHIN, HERTFORDSHIRE.—Miss Lillie Robinson has been appointed district nurse. She was trained at Crumpsall Infirmary, Manchester, and has since done private nursing at Manchester and Southport.

Hospital for Incurable Children, Hampstead.—Miss M. Allen has been appointed staff nurse. She was trained at Hampstead Poor-law Infirmary.

Infectious Diseases Hospital, Peebles, Scotland.—Miss Hattrick has been appointed matron. She was trained at the Gore Farm and the Brook Fever Hospitals, London, and has since done private work for the Chelmsford Nursing Institution. She has also had charge of the temporary hospital tents for diphtheria at De la Haye, and been charge nurse at the Fever Hospital, Yoker, near Glasgow.

Jersey General Dispensary and Infirmary.—Miss Annie Copley has been appointed matron. She was trained at the District Infirmary, Ashton-under-Lyne, and has since been ward sister at the Town's Hospital and at the Western District Hospital, Glasgow, and home sister at the General Infirmary, Gloucester. She has also done private nursing for the Oldham Nursing Association, and temporary matron's duty at the Gorleston Cottage Hospital.

Maidstone Workhouse Infirmary.—Miss Jessie E. Price has been appointed charge nurse. She was trained at Salford Union Infirmary, and has since been charge nurse at Bedford Union Infirmary.

Miller Hospital and Royal Kent Dispensary, Greenwich.—Miss Adeline M. Mace has been appointed sister. She was trained at the Royal Berkshire Hospital, Reading. She has since been nurse at the Hospital for Diseases of the Heart, Soho Square, London, and has had experience in private nursing.

Rainhill Asylum.—Miss J. A. McCallum has been appointed assistant matron. She was trained at the Victoria Hospital, Glasgow, Belvedere Fever Hospital, Glasgow, and Queen Charlotte's Hospital, London. She has since been sister at the City Hospital, Netherfield Road, Liverpool. She holds the certificate of the Central Midwives Board.

Royal Cornwall Infirmary, Truro.—Miss Emily J. Scott has been appointed matron. She has been for nine years and a half matron of Bury Hospital.

Training Ship "Exmouth" Infirmary.—Miss Maude Louise Slater has been appointed charge nurse. She was trained at Southwark Union Infirmary, and has since been head nurse and night superintendent at the same institution.

Wandsworth Infirmary.—Miss Edith M. Bradley has been appointed house sister. She was trained at the Southwark Union Infirmary, East Dulwich, and has since been sister at the Camberwell Infirmary. She has also done temporary staff duty at the West London Hospital, and private nursing.

Workhouse Infirmary, Gravelly Hill.—Miss Ada Latham has been appointed charge nurse. She was trained at the Infirmary, Fir Vale, Sheffield, where she was afterwards charge nurse. She has since done private nursing.

### To Nurses.

We invite contributions from any of our readers, and shall be glad to pay for "Notes on News from the Nursing World," "Incidents in a Nurse's Life," or for articles describing nursing experiences at home or abroad dealing with any nursing question from an original point of view, according to length. The minimum payment is 5s. Contributions on topical subjects are specially welcome. Notices of appointments, letters, entertainments, presentations, and deaths are not paid for, but we are always glad to receive them. All rejected manuscripts are returned in due course, and all payments for manuscripts used are made as early as possible after the beginning of each quarter.

### Death in our Ranks.

We regret to record the death of Nurse Clara Brereton, of Plymouth. She was trained at Toxteth Infirmary, Liverpool, and had done several years' private nursing in the West of England as a member of the West of England Nurses' Co-operation at Plymouth. She went home to nurse a cousin suffering from enteric fever, which she contracted, and subsequently died last month.

### Novelties for Nurses.

(By Our Shopping Correspondent.)

### RUDGE-WHITWORTH BICYCLES.

When a district nurse requires a bicycle she naturally wishes to secure a machine that will stand the strain which riding in all weathers and over bad roads must put upon it. Only the best bicycles are suitable for constant use, and a Rudge-Whitworth bicycle fulfils this description. Rudge-Whitworth bicycles are elegant machines, and, moreover, extremely moderate in cost. The "Crescent" bicycle can be bought for only £6 12s., and is a sound and useful machine, with freewheel and two brakes. It is even cheaper still with fixed wheel and one brake. The "Standard" and "Special" are both excellent at a higher price, and the "Aero-Special" is a charming bicycle de luxe, which, with every improvement, is only £14 5s. Messrs. Rudge-Whitworth allow payment by instalment, or a cash discount for immediate payment. They have branches in most large towns and three depots in London—at 230 Tottenham Court Road, 23 Holborn Viaduct, E.C., and 160 Regent Street, W.

### TRAVEL NOTES AND QUERIES.

By our Travel Correspondent.

A Holiday at Malvern (F.E.S.).—I am very glad your holiday was so successful last year. I fear there is nothing at Malvern that would come within your means. I could give you an address in North Cornwall and in North Devon, but not Dartmoor. Write (using my name) to Miss Snell, Ivy Cottage, Caerhays, near St. Austell, Cornwall. Her terms are very moderate, and the country is lovely. In the tourist season you can have an excursion ticket for a fortnight for 26s., and there is 7s. for a carriage from the station. Also use my name in writing to Mrs. Alan Murray, Brookside, Croyde, North Devon. In both cases write early, as these houses soon fill.

A Holiday for Five People from Swanage (Bee). I shall be most pleased if I can help in your kind plan, but please give me some particulars. The number to be provided for makes the amount to be spent heavy. Tell me exactly how much money you can afford to spend, also whether you would like to go abroad, because the journey to Belgium would be very cheap; only 9s. second-class return. See answer to "F.E.S." Would the first mentioned address suit you? The second would not, as there is not sufficient accommodation. Tell me the date when you wish to take your holiday.

Rules in Regard to Correspondence for this Section.—All questioners must use a pseudonym for publication, but the communication must also bear the writer's own name and address as well, which will be regarded as confidential. All such communications to be addressed "Travel Correspondent, 28 Southampton Street, Strand." No charge will be made for inserting and answering questions in the inquiry column, and all will be answered in rotation as space permits. If an answer by letter is required, a stamped and addressed envelope must be enclosed, together with 2s. 6d., which fee will be devoted to the objects of "The Hospital" Convalescent Fund. Ten days must be allowed before an answer can be published.

### Notes and Queries.

**REGULATIONS.**

**The Editor is always willing to answer in this column, without any fee, all reasonable questions, as soon as possible.**

**But the following rules must be carefully observed.**

1. **Every communication must be accompanied by the name and address of the writer.**
2. **The question must always bear upon nursing, directly or indirectly.**

**If an answer is required by letter a fee of half-a-crown must be enclosed with the note containing the inquiry.**

*Hospital Maid.*

(79) As a hospital maid with a Board School education, can I become a nurse? *Anxious No. 4.*

Certainly. Write to the Middlesex Hospital, and get "How to Become a Nurse" from any bookseller, or from The Scientific Press, 28 Southampton Street, Strand.

*A Matron's Obligations.*

(80) Has a matron control over her nurses when off duty outside the hospital, and if she disapproved, might the certificate be affected?—*Anxious No. 3.*

Certainly a matron should have a moral control over her nurses at all times, and she should explain to them that any unseemly action on their part reflects on their hospital. The conduct of a nurse affects the certificate.

*How to Become a Nurse.*

(81) Will you give me information how to set about becoming a nurse?—*Maria G.*

"How to Become a Nurse," which you can purchase for 2s. at The Scientific Press, 28 Southampton Street, Strand, gives you all the information you require.

*Text-books.*

(82) Can you tell me what I shall have to spend on text-books during my training as a nurse?—*E. M. C.*

This is a question which cannot be answered definitely. If your training school does not require you to buy special books, Dr. Watson's two books on nursing and midwifery are all that you need, and these can be obtained at The Scientific Press for about 12s.

*Sanitary Inspector.*

(83) Where can I obtain information respecting sanitary inspectorships? I am a trained nurse; would this be in my favour?—*G. G.*

Write to the Royal Sanitary Institute, Margaret Street. Your training should help you to acquire the necessary knowledge more easily than without it.

*Medico-Psychological Certificate.*

(84) How can I, a mental nurse, obtain the Medico-Psychological certificate?—*Anxious One.*

Write to the Secretary of the Medico-Psychological Association, 11 Chandos Street, W.

*Nurses in Glasgow.*

(85) Can you give me the address of a nurses' home in Glasgow?—*L. M. T.*

Glasgow Sick and Poor Private Nursing Association, 218 Bath Street.

*Army Nursing.*

(86) I have a certificate from a children's hospital. What else must I obtain to become an Army nurse or some other post?—*Myra.*

Unfortunately your training will not help you. You must have three years' training in a general hospital to obtain an Army or any other good position.

*Consumptive Sanatorium.*

(87) Can you tell me of a high-class sanatorium for a youth to whom expense is no object?—*Anxious.*

You will find a full list of all the best Sanatoria in "Medical Homes," which you can obtain for 7d., post free, from The Scientific Press, 28 and 29 Southampton Street, Strand, London, W.C.

**Handbooks for Nurses.**

| | Post Free. |
|---|---|
| "A Handbook for Nurses." (Dr. J. K. Watson) ... | 5s. 4d. |
| "Nurses' Pronouncing Dictionary of Medical Terms" | 2s. 0d. |
| "Art of Massage." (Creighton Hale.) ... ... ... | 6s. 0d. |
| "Surgical Bandaging and Dressings." (Johnson Smith.) ... ... ... ... ... ... | 2s. 0d. |
| "Hints on Tropical Fevers." (Sister Pollard.) ... | 1s. 8d. |

Of all booksellers or of The Scientific Press, Limited, 28 & 29 Southampton Street, Strand, London, W.C.

### For Reading to the Sick.

WITH CHRIST.

"Before that Light one grows to such content
That to turn back from it to aught beside
The soul can never possibly consent;
Seeing that the good, by which is satisfied
Our will, is centred there; outside that rest;
Defect attends what perfect there doth bide."
*Dante.*

To be in heaven is to pass eternity "with Christ," under whose loving rule all things truly human, as well as all things that are Divine, shall flourish and abound. For ever since His reign on earth has begun, though not as yet thoroughy acknowledged everywhere, civilisation has progressed, with its learning, its freedom, its order, its political life, its arts and sciences, its security and joy and brightness and vigour. In short, what has created the civilised world but the rule of Christ the King? And when Christ the King rules without let or hindrance, not only "the earth may be glad thereof, yea, the multitude of the isles may be glad thereof," but He will be a Human Centre as well as a Divine Tower; a Human as well as a Divine Lord.

And when we see Him, surely we shall see the Incarnation of all that we admire upon earth! Will He not be the Embodiment—the realised Ideal—of courage, of purity, of love, of gentleness, of knowledge, of intellectual power—of all that is beautiful and fair?

Is He not, already, the Light of this world, as well as of the City of God? For what is more beautiful, here on earth, than the character impressed with the seal of Jesus Christ? —whether it be in the little child, or in the grown-up man or woman, or in the "ancient men"? What is it that gives a glory to any personal character among us now, but some trait or other of the character of our Lord—some ray or other caught from the glory round His Head?—*Bishop Webb.*

What will it be to see the Glorified Manhood of our Divine Redeemer; to see His Face, shining above the brightness of the sun, yet in rays of love; to see that look which brought us to ourselves and Him; to hear the Voice of love, which called us and we followed Him; to see all bright and pure and radiant with light and Divine lustre, the glorified scars which for us and for our salvation He received, and which ever since have in the presence of the Eternal Father been pleading witnesses of His Love and Suffering for our guiltiness, withholding His anger from us; and in that sight, "to have our heart and all our senses filled with His Glory and Love. . . . And because no created heart can contain such love, we shall joy in the bliss of others as our own. In all we shall behold, in all love God. The praise of all shall gladden us; each voice, which has learned the new song, shall swell, with its own special beauty, the everlasting harmony; the glory of each several star in that more blessed firmament shall shed its own special lustre. While all joy in those "unfailing streams of Mercy, Goodness, Majesty, and Love, and sweet Peace," which shall issue forth from the Redeemer, "all shall encompass Him with longings of love, and shall render all thanksgiving and praise," thanking Him that they are His and of Him, and casting their crowns before Him, joying most of all that "they are justified by His Grace, redeemed by His Blood, saved by His Love, glorified by His Merits."—*E. B. P.*

## Figures and Tables

**Figure f1:**
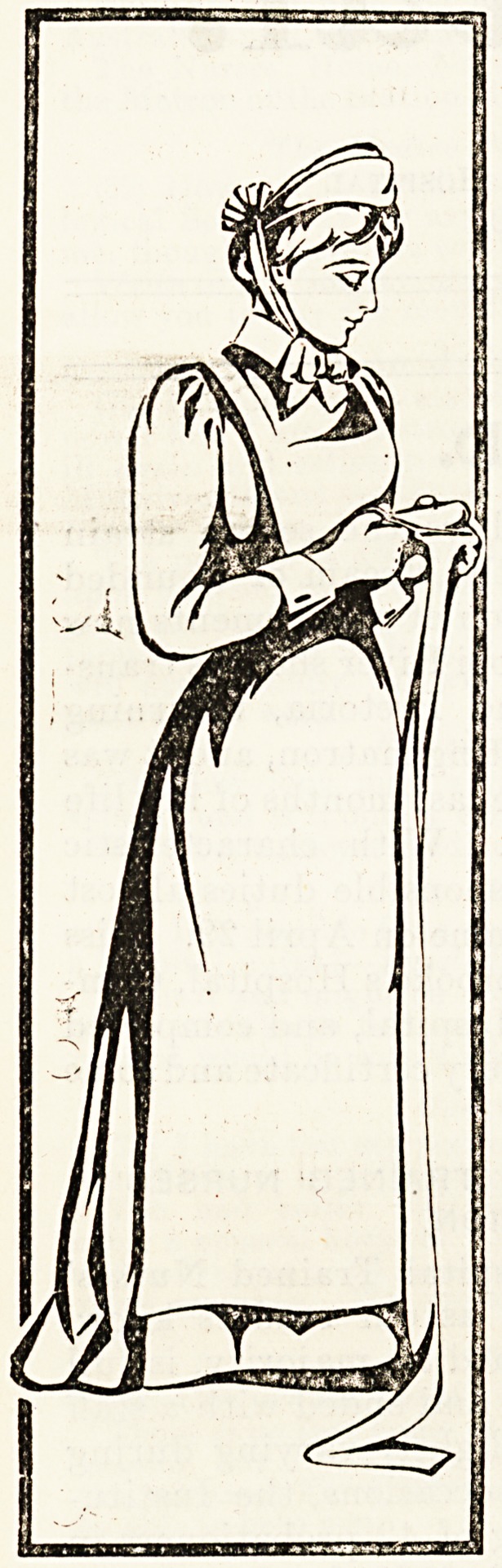


**Figure f2:**